# The effects of dairy on the gut microbiome and symptoms in gastrointestinal disease cohorts: a systematic review

**DOI:** 10.1017/gmb.2024.2

**Published:** 2024-05-07

**Authors:** Clíona Ní Chonnacháin, Emma L. Feeney, Clare Gollogly, Denis C. Shields, Christine E. Loscher, Paul D. Cotter, Nessa Noronha, Roisin Stack, Glen A. Doherty, Eileen R. Gibney

**Affiliations:** 1 Food for Health Ireland, University College Dublin, Dublin, Ireland; 2 Institute of Food and Health, University College Dublin, Dublin, Ireland; 3 Conway Institute of Biomolecular and Biomedical Research, University College Dublin, Dublin, Ireland; 4 School of Medicine and Medical Science, University College Dublin, Dublin, Ireland; 5 School of Biotechnology, Dublin City University, Dublin, Ireland; 6 Department of Food Biosciences, Teagasc Food Research Centre, APC Microbiome Ireland and VistaMilk, Dublin, Ireland; 7 Centre for Colorectal Disease, St Vincent’s University Hospital, Dublin, Ireland

**Keywords:** gastrointestinal microbiome, dairy, inflammatory bowel disease, functional gastrointestinal disorder, fermented foods, gut health

## Abstract

Bovine dairy foods provide several essential nutrients. Fermented bovine dairy foods contain additional compounds, increasing their potential to benefit gastrointestinal health. This review explores the effects of dairy consumption on the gut microbiome and symptoms in gastrointestinal disease cohorts. Human subjects with common gastrointestinal diseases (functional gastrointestinal disorders and inflammatory bowel disease) or associated symptoms, and equivalent animal models were included. A systematic literature search was performed using PubMed, Embase and Web of Science. The search yielded 3014 studies in total, with 26 meeting inclusion criteria, including 15 human studies (1550 participants) and 11 animal studies (627 subjects). All test foods were fermented bovine dairy products, primarily fermented milk and yogurt. Six studies reported increases in gastrointestinal bacterial alpha diversity, with nine studies reporting increases in relative *Lactobacillus* and *Bifidobacterium* abundance. Six studies reported increases in beneficial short-chain fatty acids, while three reported decreases. Gastrointestinal symptoms, specifically gut comfort and defecation frequency, improved in 14 human studies. Five animal studies demonstrated reduced colonic damage and improved healing. This review shows fermented bovine dairy consumption may improve gut microbial characteristics and gastrointestinal symptoms in gastrointestinal disease cohorts. Further human intervention studies are needed, expanding test foods and capturing non-self-reported gastrointestinal measures.

## Introduction

Bovine dairy foods provide a wide range of essential nutrients, including bioavailable amino acids, fats, calcium, phosphorus, and several vitamins (Haug et al., 2007). These nutrients contribute significantly to musculoskeletal growth and maintenance, and general well-being (Thorning et al., 2016). A recent data modelling study demonstrated that milk (bovine) is the main contributing food item to the global nutrient availability of calcium, vitamin B2, lysine, and dietary fat, emphasising the role of dairy in the modern diet (Smith et al., 2022). Dairy foods are widely accessible, and a wide variety of food types are available, including milk, butter, cream, and fermented dairy foods such as cheese, yogurt, and kefir (Haug et al., 2007). Fermented dairy foods are produced through the desirable action of microorganisms (Marco et al., 2021). This process can enhance the nutritional quality of dairy foods, potentially providing probiotics (live microorganisms), prebiotics (substrates for desirable gut microbes), and additional bioactive compounds (Hill et al., 2014; Davani-Davari et al., 2019). These attributes have the potential to increase gut microbial diversity and improve aspects of digestive, cardiovascular, and metabolic health, thus, fermented dairy foods can provide health benefits beyond the scope of non-fermented dairy (Leeuwendaal et al., 2022).

Gastrointestinal complications are widely experienced, with a 2021 study showing approximately 40% of the global population experience at least one symptom associated with functional gastrointestinal disorders (FGIDs) (Sperber et al., 2021). FGIDs cover a range of gastrointestinal tract disorders, encompassing symptoms such as constipation, diarrhoea, bloating, and abdominal pain (Sperber et al., 2021). Irritable bowel syndrome (IBS) is a common FGID, with a 2021 study showing worldwide prevalence (as per Rome III criteria) is approximately 10% (Sperber et al., 2021). FGIDs and associated symptoms can severely affect quality of life and are burdensome on healthcare systems (Sperber et al., 2021). Gastrointestinal symptoms associated with FGIDs (e.g., diarrhoea, abdominal pain) are also experienced in clinically defined gastrointestinal diseases. Specifically, inflammatory bowel disease (IBD) is a chronic condition primarily affecting the lower gastrointestinal tract (Xavier and Podolsky, 2007). IBD encompasses both Crohn’s disease (CD) and ulcerative colitis (UC), which are characterised by chronic gastrointestinal inflammation (Xavier and Podolsky, 2007). UC is localised to the colon, while inflammation can occur anywhere along the GI tract in CD (Gohil and Carramusa, 2014). A 2017 review reported global IBD prevalence as over 6.8 million (95% UI 6.4–7.3) cases (Wang et al., 2023). In 2020, global CD and UC prevalence were reported as 3 to 20 and 1 to 24 cases per 100,000, respectively (Feuerstein and Cheifetz, 2017; Du and Ha, 2020). Gastrointestinal symptoms can be managed through medical strategies and lifestyle modifications in FGIDs and IBD, and thus, it is important to understand how dietary intake can influence the parameters of gastrointestinal health in these cohorts (Fikree and Byrne, 2021).

The gut microbiome plays an important role in human health, wherein the combined microbial community, or specific components thereof, can, depending on the composition and/or function, benefit the host (Ogunrinola et al., 2020). The gut microbiome is involved in the maintenance of gastrointestinal health as well as aspects of immune, metabolic, and mental functions (Valdes et al., 2018). The gut microbial environment is influenced by a wide range of factors including age, lifestyle, and genetics (Ogunrinola et al., 2020). Dietary intake is a strong predictor of gut microbial composition, and therefore understanding gut microbial responses to foods is important (Hasan and Yang, 2019). Gut microbial dysbiosis is defined as perturbations to the structure of complex commensal communities in the gut (Petersen and Round, 2014). Dysbiosis in the gut microbiota is characterised by reduced diversity, expansion of pathobionts (organisms that can be harmful under certain conditions), and loss of beneficial microbes (Petersen and Round, 2014; Jochum and Stecher, 2020).

While the pathogenesis of FGIDs and IBD is complex, gut microbial dysbiosis appears to be intertwined with such gastrointestinal diseases and disorders (Xavier and Podolsky, 2007; Holtmann et al., 2016). In comparison to healthy individuals, FGID and IBD cohorts have been shown to have different gut microbial characteristics (Duan et al., 2019; Pittayanon et al., 2020; Wang et al., 2020; Clooney et al., 2021; Abdel-Rahman and Morgan, 2022; Kim et al., 2023). A 2019 systematic review of 16 studies showed IBS patients had lower faecal bacterial alpha diversity, compared to healthy controls (Duan et al., 2019). A 2020 meta-analysis of 23 case–control studies showed IBS patients had lower faecal *Lactobacillus* and *Bifidobacterium*, and higher *Escherichia coli*, relative to healthy controls (Wang et al., 2020). However, a more recent review of 16 studies focusing on longitudinal omics studies only, showed significant heterogeneity across gut microbial characteristics in IBS cohorts across studies, concluding that defining uniform gut microbial characteristics of an IBS-related gut microbiota is challenging (Ng et al., 2023). However, while clearer characterisation of IBS-related gut microbial characteristics is needed, overall, gut microbial dysbiosis is prevalent in this cohort (Wang et al., 2020; Kim et al., 2023; Ng et al., 2023). In IBD patients, a recent meta-analysis of 13 studies showed faecal bacterial alpha diversity was lower compared to healthy controls, and this was more pronounced in CD compared to UC (Abdel-Rahman and Morgan, 2022). Similarly to studies in IBS cohorts, studies comparing gut microbial taxa of healthy cohorts to IBD cohorts also had heterogenous methods and results, although Pittayanon et al. (2020) reported some notable differences in bacterial taxa between healthy, CD and UC cohorts, based on a review of 45 studies. Thus, overall, gut microbial dysbiosis is prevalent among FGID and IBD cohorts, but it should be noted that further studies are needed to determine distinctive gut microbial characteristics in such cohorts (Duan et al., 2019; Pittayanon et al., 2020; Wang et al., 2020; Clooney et al., 2021; Abdel-Rahman and Morgan, 2022; Kim et al., 2023).

Dairy foods provide a range of nutrients, with certain fermented dairy foods also providing probiotics, prebiotics, and bioactive compounds (Haug et al., 2007). Therefore, dairy has the potential to influence the gut microbiome and gastrointestinal health, particularly in individuals with gastrointestinal complications. Identification of dairy foods that could improve common gastrointestinal symptoms and ameliorate gut microbial dysbiosis among FGID and IBD cohorts would be beneficial, as dairy consumption may be an accessible method of improving gastrointestinal health in such cohorts. This review aims to provide a comprehensive synthesis of intervention studies examining the effects of bovine dairy consumption on the gut microbiome and gastrointestinal health outcomes in human and animal (porcine and murine) cohorts with FGIDs, IBD, and associated symptoms.

## Methods

### Literature search

The protocol for this review was registered on PROSPERO (Registration ID: CRD42023392814) and follows PRISMA (Preferred Reporting Items for Systematic reviews and Meta-Analyses) guidelines (Moher et al., 2010). A search strategy was developed based on population, intervention, comparator, and outcome (PICO) parameters. Inclusion criteria for the types of participants, interventions, controls, and outcomes are outlined in the PICO framework (Table 1). Populations included were human adults with gastrointestinal diseases or symptoms, and equivalent porcine and murine models. Gastrointestinal disease refers to IBD (UC and CD), FGIDs, and their associated gastrointestinal symptoms. Gastrointestinal symptoms refer to any symptoms related to the lower gastrointestinal tract, such as bloating, gas, diarrhoea, and constipation. The scope of this review focuses on gastrointestinal symptoms (e.g., diarrhoea, abdominal pain, bloating) and disease status in IBD. Many of the gastrointestinal symptoms associated with IBD are also experienced in FGIDs and therefore, these populations were also included to extend the search. Animal models were included as they allow more invasive methods of gastrointestinal analysis, which adds to the review by providing non-subjective measures of gastrointestinal health. Animal models were restricted to porcine and murine as they are considered physiologically relevant to humans, with respect to gastrointestinal research (Mizoguchi, 2012; Gonzalez et al., 2015). Interventions included dairy intake, which includes bovine dairy in any form (e.g., whole-milk, yogurt, whey). Comparators accepted were alternative dairy foods, dairy restriction, standard diets, or healthy cohorts. The outcomes included changes in gastrointestinal disease status, gastrointestinal symptoms, gut microbial characteristics (bacterial diversity and relative bacterial abundance), and faecal short-chain fatty acid (SCFA) concentrations. Inclusion criteria also included studies published in English, randomised-controlled dietary intervention trials, and controlled dietary intervention trials for human and animal studies, respectively. The search strategy was then used in three databases to identify relevant studies: PubMed, Embase, and Web of Science (from journal inception to December 2022). See supplementary material for the extended search strategy.

**Table 1.** PICO criteria

Parameter	Criteria
Population	Human adults (>18y) with gastrointestinal diseases/disorders* or symptoms Animal (porcine or murine) models for gastrointestinal disease/disorders or symptoms
Intervention	Bovine dairy consumption (e.g., milk, yogurt, cheese, kefir, whey)
Comparator	Alternative dairy food (e.g., non-fermented milk) Dairy restriction Standard diet Healthy cohort
Outcome	Change in gastrointestinal disease status (clinical) Change in gastrointestinal symptom status (self-reported) Change in gut microbial characteristics (relative bacterial abundance OR bacterial diversity) Change in SCFA concentration

Abbreviations: PICO, population, intervention, control, outcome; SCFA, short-chain fatty acid.

* Refers to inflammatory bowel disease, functional gastrointestinal disorders, and their associated gastrointestinal symptoms.

### Data collection and screening

Search results from each database were downloaded and exported into Endnote (Clarivate Analytics, PA, USA). References from each database were merged and duplicates were removed. Studies were then imported into Covidence for screening against selection criteria by title, abstract, and then full text (Covidence systematic review software, Veritas Health Innovation, Melbourne, Australia). Two authors (CNC, CG) independently completed the screening process to select the final studies meeting the inclusion criteria. Where discrepancies arose, a third author (ERG) was introduced to resolve disagreements.

### Data extraction and analysis

A data extraction form was used to collect study data. Variables considered for extraction included study design, study setting, population characteristics (e.g., human IBD cohort, murine IBD model), test food (e.g., fermented milk, yogurt), control (e.g., PBS, healthy cohort), intervention dose (e.g., grams per day, grams per kg body weight), intervention duration, analysis methods (e.g., questionnaire, faecal metagenomic analysis) and results (e.g., gut microbial composition, diarrhoea frequency). One author completed the data extraction process independently (CNC) and the second author (CG) cross-checked the data extraction form.

### Risk of bias assessment

The Cochrane ‘Risk of bias’ 2.0 tool was used to assess the risk of bias (RoB) in the human studies meeting inclusion criteria (Sterne et al., 2019). This tool assesses RoB based on five domains: risk of bias arising from randomisation, deviations from the intended interventions, missing outcome data, measurement of the outcome, and selection of the reported result. For the animal studies meeting inclusion criteria, SYRCLE’s RoB tool was used to assess bias (Hooijmans et al., 2014). The tool assesses RoB based on five domains: risk of bias arising from selection, performance, detection, attrition, and reporting (Hooijmans et al., 2014). Risk of bias assessments were carried out by two reviewers (CNC, CG), and discrepancies were addressed through discussion.

The studies meeting inclusion criteria were grouped by population type (human or animal) to synthesise the results. Within population types, studies were further grouped by outcome (gut microbiome/SCFAs or gastrointestinal health parameters/symptoms). A narrative synthesis of the respective results from each group of studies was then conducted.

## Results

**Figure 1.**
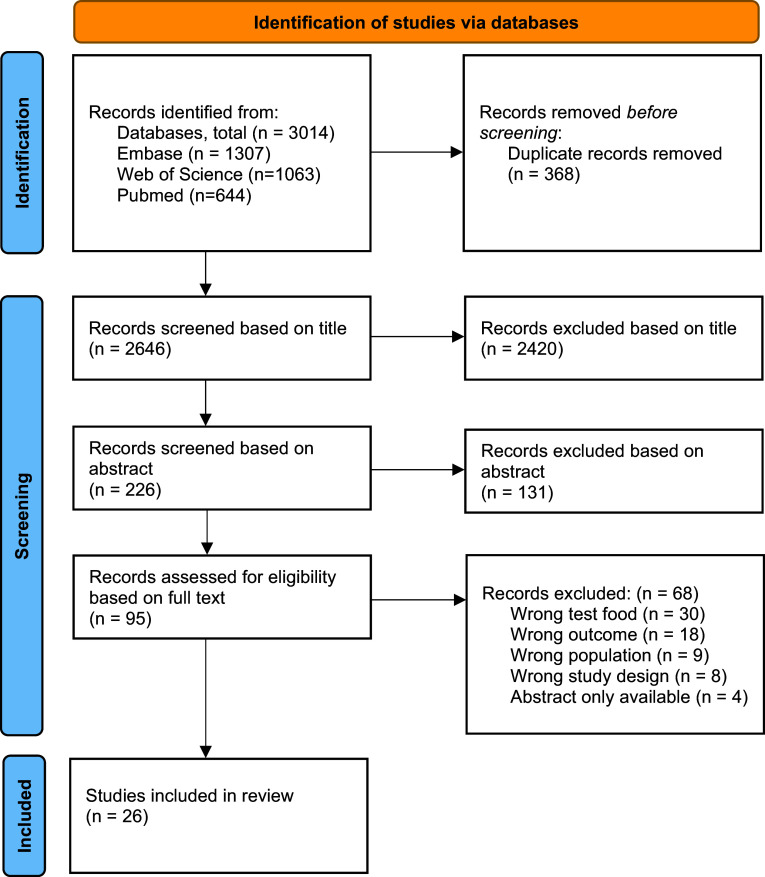
PRISMA flow diagram.

The search strategy identified a total of 2646 de-duplicated studies. After the overall screening process, 26 studies were considered eligible for the review and were included in the data synthesis. See Figure 1 for the PRISMA flow diagram providing further details of the search results and screening process. Most studies (*n =* 2420) were excluded at the title screening phase. The primary reasons for exclusion at the title screening phase were test foods (e.g., non-bovine milks including sheep’s milk and human milk, probiotic strains alone, prebiotics alone), outcomes (e.g., effects on hypertension, adiposity, inflammatory response, colon cancer) or population groups which were out of scope (e.g., diabetic cohorts, lactose intolerant cohorts, paediatric cohorts, non-murine/porcine animal cohort). The main reason for exclusion at the full-text screening phase was due to test foods that were out of scope (*n =* 30), followed by outcomes (*n =* 18) and population types (*n =* 9) that failed to meet inclusion criteria (Figure 1).

### Study details

Fifteen studies within human populations were identified (Supplementary Table S1), with a total of 1550 participants across the studies (Beniwal et al., 2003; Ishikawa et al., 2003; Kato et al., 2004; Matsumoto et al., 2010; Søndergaard et al., 2011; Thijssen et al., 2011; Marteau et al., 2013; Tilley et al., 2014; Veiga et al., 2014; Gomi et al., 2015; Liu et al., 2015; Le Nevé et al., 2019; Yilmaz et al., 2019; Li et al., 2020; Mokhtar et al., 2021). Studies were conducted from 2003 to 2021 with the majority taking place in Asia (*n =* 7) (Ishikawa et al., 2003; Kato et al., 2004; Matsumoto et al., 2010; Gomi et al., 2015; Liu et al., 2015; Li et al., 2020; Mokhtar et al., 2021) and Europe (*n =* 7) (Søndergaard et al., 2011; Thijssen et al., 2011; Marteau et al., 2013; Tilley et al., 2014; Veiga et al., 2014; Le Nevé et al., 2019; Yilmaz et al., 2019). Sample sizes ranged from 20 to 530 participants and ages ranged from 18 to 94 years (Beniwal et al., 2003; Ishikawa et al., 2003; Kato et al., 2004; Matsumoto et al., 2010; Søndergaard et al., 2011; Thijssen et al., 2011; Marteau et al., 2013; Tilley et al., 2014; Veiga et al., 2014; Gomi et al., 2015; Liu et al., 2015; Le Nevé et al., 2019; Yilmaz et al., 2019; Li et al., 2020; Mokhtar et al., 2021). Seven studies involved participants with FGIDs (diarrhoea, constipation, or general digestive symptoms) (Beniwal et al., 2003; Matsumoto et al., 2010; Marteau et al., 2013; Tilley et al., 2014; Gomi et al., 2015; Liu et al., 2015; Li et al., 2020), five studies included IBS patients (Søndergaard et al., 2011; Thijssen et al., 2011; Veiga et al., 2014; Le Nevé et al., 2019; Mokhtar et al., 2021) and three studies included IBD patients (Ishikawa et al., 2003; Kato et al., 2004; Yilmaz et al., 2019). Gastrointestinal symptoms and disease criteria included both clinical diagnosis (e.g., Rome criteria) and self-reported digestive health problems (e.g., self-reported mild constipation) (Supplementary Table S1) (Beniwal et al., 2003; Ishikawa et al., 2003; Kato et al., 2004; Matsumoto et al., 2010; Søndergaard et al., 2011; Thijssen et al., 2011; Marteau et al., 2013; Tilley et al., 2014; Veiga et al., 2014; Gomi et al., 2015; Liu et al., 2015; Le Nevé et al., 2019; Yilmaz et al., 2019; Li et al., 2020; Mokhtar et al., 2021).

Eleven studies within animal populations were identified, with a total of 627 subjects reported across the studies (Supplementary Table S2) (Uchida and Mogami, 2005; Sprong et al., 2010; Veiga et al., 2010; Lee et al., 2015; Liu et al., 2017; Sevencan et al., 2019; Rabah et al., 2020; Yan et al., 2020; Zhang et al., 2020; Feng et al., 2022; Yang et al., 2022). Studies were conducted between 2005 and 2022 with the majority, like the human studies reported above, taking place in Asia (*n =* 6) (Uchida and Mogami, 2005; Liu et al., 2017; Yan et al., 2020; Zhang et al., 2020; Feng et al., 2022; Yang et al., 2022) and Europe (*n =* 3) (Sprong et al., 2010; Sevencan et al., 2019; Rabah et al., 2020). Sample sizes ranged from 31 to 144 animal participants, aged between 1 to 18 weeks (Uchida and Mogami, 2005; Sprong et al., 2010; Veiga et al., 2010; Lee et al., 2015; Liu et al., 2017; Sevencan et al., 2019; Rabah et al., 2020; Yan et al., 2020; Zhang et al., 2020; Feng et al., 2022; Yang et al., 2022). Seven studies included mice (Veiga et al., 2010; Lee et al., 2015; Liu et al., 2017; Rabah et al., 2020; Yan et al., 2020; Zhang et al., 2020; Yang et al., 2022) and four studies included rats (Uchida and Mogami, 2005; Sprong et al., 2010; Sevencan et al., 2019; Feng et al., 2022). Of these, ten standard murine species including Wistar rats or C57BL6 mice were used (Uchida and Mogami, 2005; Sprong et al., 2010; Lee et al., 2015; Liu et al., 2017; Sevencan et al., 2019; Rabah et al., 2020; Yan et al., 2020; Zhang et al., 2020; Feng et al., 2022; Yang et al., 2022). Gastrointestinal complications in these animals were chemically induced by the administration of dextran sodium sulphate (*n =* 6), trinitrobenzene sulfonic acid (*n =* 2), loperamide (*n =* 1) or antibiotics (*n =* 1) (Uchida and Mogami, 2005; Sprong et al., 2010; Lee et al., 2015; Liu et al., 2017; Sevencan et al., 2019; Rabah et al., 2020; Yan et al., 2020; Zhang et al., 2020; Feng et al., 2022; Yang et al., 2022). Alternatively, Veiga et al. (2010) used TRUC mice species (TNFR1/p55^−/−^), a genetic model for UC (Supplementary Table S2).

### Study design and methods

Table 2 outlines the study design and methods used in human studies. The majority (*n =* 11) of test foods were fermented milk (Ishikawa et al., 2003; Kato et al., 2004; Matsumoto et al., 2010; Søndergaard et al., 2011; Thijssen et al., 2011; Marteau et al., 2013; Tilley et al., 2014; Veiga et al., 2014; Gomi et al., 2015; Le Nevé et al., 2019; Mokhtar et al., 2021), three studies examined yogurt consumption (Beniwal et al., 2003;Liu et al., 2015; Li et al., 2020), and Yilmaz et al. (2019) investigated kefir consumption. Thus, all test foods included were fermented dairy foods. No study with a non-fermented dairy food (e.g., whole milk) met the study inclusion criteria. Of the fermented milk, seven studies investigated mixed-strain fermented milk, three studies investigated *Lactobacillus casei* strain Shirota fermented milk, and one study investigated *Lactobacillus* fermented milk (Ishikawa et al., 2003; Kato et al., 2004; Matsumoto et al., 2010; Søndergaard et al., 2011; Thijssen et al., 2011; Marteau et al., 2013; Tilley et al., 2014; Veiga et al., 2014; Gomi et al., 2015; Le Nevé et al., 2019; Mokhtar et al., 2021). Controls were mostly non-fermented or acidified milk (*n =* 9) (Matsumoto et al., 2010; Søndergaard et al., 2011; Thijssen et al., 2011; Marteau et al., 2013; Tilley et al., 2014; Veiga et al., 2014; Gomi et al., 2015; Liu et al., 2015; Le Nevé et al., 2019), or deprivation (i.e., meaning the removal of a dairy food from the diet) (*n =* 3) (Beniwal et al., 2003; Ishikawa et al., 2003; Yilmaz et al., 2019). Three studies provided nutritional information for test foods (*n =* 2 fermented milk, *n =* 1 yogurt), which is outlined in Supplementary Table S3 (Matsumoto et al., 2010; Tilley et al., 2014; Liu et al., 2015). Fat contents ranged from <0.01 g to 2.91 g per 100 g, protein contents ranged from 1.25 to 2.73 g per 100 g and carbohydrate contents ranged from 11.75 to 18.00 g/100 g (Matsumoto et al., 2010; Tilley et al., 2014; Liu et al., 2015). Li et al. (2020) and Mokhtar et al. (2021) included healthy cohorts free of gastrointestinal disease as control groups. Trial duration ranged from 1 week to 1 year and test food quantities consumed per day ranged from 65 mL to 500 mL (Beniwal et al., 2003; Ishikawa et al., 2003; Kato et al., 2004; Matsumoto et al., 2010; Søndergaard et al., 2011; Thijssen et al., 2011; Marteau et al., 2013; Tilley et al., 2014; Veiga et al., 2014; Gomi et al., 2015; Liu et al., 2015; Le Nevé et al., 2019; Yilmaz et al., 2019; Li et al., 2020; Mokhtar et al., 2021). Gastrointestinal disease status and symptoms were assessed through self-reported symptom questionnaires and disease-specific questionnaires (e.g., IBS Symptom Severity Scale) (Beniwal et al., 2003; Matsumoto et al., 2010; Søndergaard et al., 2011; Thijssen et al., 2011; Marteau et al., 2013; Tilley et al., 2014; Gomi et al., 2015; Liu et al., 2015; Le Nevé et al., 2019; Yilmaz et al., 2019; Li et al., 2020; Mokhtar et al., 2021). In addition to questionnaires, Ishikawa et al. (2003) and Kato et al. (2004) performed colonoscopies to determine gastrointestinal disease status. Gut microbiota was assessed using polymerase chain reaction (PCR) based techniques (*n =* 4) (Matsumoto et al., 2010; Liu et al., 2015; Le Nevé et al., 2019; Yilmaz et al., 2019), DNA or 16S rRNA sequencing (*n =* 2) (Veiga et al., 2014; Liu et al., 2015) or culturing methods (*n =* 2) (Ishikawa et al., 2003; Kato et al., 2004). SCFAs were analysed by high-performance liquid chromatography (*n =* 3) (Ishikawa et al., 2003; Kato et al., 2004; Matsumoto et al., 2010) gas chromatography (*n =* 2) (Liu et al., 2015; Li et al., 2020), or *in vitro* methods (*n =* 1) (Veiga et al., 2014). Eleven of 15 studies specified their primary outcome (*n =* 3) (Ishikawa et al., 2003; Kato et al., 2004; Le Nevé et al., 2019) or had just one outcome (*n =* 8) (Beniwal et al., 2003; Søndergaard et al., 2011; Thijssen et al., 2011; Marteau et al., 2013; Tilley et al., 2014; Veiga et al., 2014; Gomi et al., 2015; Liu et al., 2015). Of these, most stated gastrointestinal symptoms (*n =* 8) (Beniwal et al., 2003; Søndergaard et al., 2011; Thijssen et al., 2011; Marteau et al., 2013; Tilley et al., 2014; Gomi et al., 2015; Liu et al., 2015; Le Nevé et al., 2019) or gastrointestinal disease status (*n =* 2) (Ishikawa et al., 2003; Kato et al., 2004) as their primary outcome. Veiga et al. (2014) stated changes in gut microbial characteristics as their primary outcome. Four studies with multiple outcomes did not specify a primary outcome (Matsumoto et al., 2010; Yilmaz et al., 2019; Li et al., 2020; Mokhtar et al., 2021).

**Table 2.** Methods (human studies)

Author	Year	Test food	Quantity (per day)	Control	Trial length	Outcome* (method)
Ishikawa et al. (2003)	2003	MSFM	100 mL	Deprivation	1 year	**GID** (colonoscopy, questionnaire) GM (culturing) SCFAs (HPLC)
Beniwal et al. (2003)	2003	Yogurt	227 g	Deprivation	8 weeks	**GIS** (questionnaire)
Kato et al. (2004)	2004	MSFM	100 mL	FM**	12 weeks	**GID** (colonoscopy, questionnaire) GM (culturing) SCFAs (HPLC)
Matsumoto et al. (2010)	2010	LcS FM	80 mL	NFM	4 weeks	GIS (questionnaire) GM (qPCR) SCFAs (HPLC)
Søndergaard et al. (2011)	2011	MSFM	500 mL	AM	8 weeks	**GIS** (questionnaire)
Marteau et al. (2013)	2013	MSFM	125 g	AM	4 weeks	**GIS** (questionnaire)
Tilley et al. (2014)	2014	LcS FM	65 mL	NFM	8 weeks	**GIS** (questionnaire)
Veiga et al. (2014)	2014	MSFM	250 g	AM	4 weeks	**GM** (NGS) SCFAs (in vitro)
Gomi et al. (2015)	2015	MSFM	100 mL	NFM	2 weeks	**GIS** (questionnaire)
Liu et al. (2015)	2015	Yogurt	110 mL	NFM	7 weeks	**GIS** (questionnaire) GM (qPCR) SCFAs (GC)
Thijssen et al. (2011)	2016	LcS FM	130 mL	NFM	8 weeks	**GIS** (questionnaires)
Le Nevé et al. (2019)	2019	MSFM	150 g	NFM	2 weeks	**GIS** (questionnaires) GM (qPCR) GM FC (H2, CH2 breath concentrations)
Yilmaz et al. (2019)	2019	Kefir	400 mL	Deprivation	4 weeks	GIS (questionnaires) GM (RT-qPCR)
Li et al. (2020)	2020	Yogurt	250 mL	Healthy cohort	1 week	GM (16S PCR) SCFAs (GC)
Mokhtar et al. (2021)	2021	LFM	375 mL	Healthy cohort	30 days	GIS (questionnaire) ITT (food colourant self-reported)

Abbreviations: AM, acidified milk; GC, gas chromatography; GID, gastrointestinal disease; GIS, gastrointestinal symptoms; GM, gut microbiota; GM FC, gut microbial functional capacity; ITT, intestinal transit time; LcS, *Lactobacillus casei* strain Shirota; LFM, *Lactobacillus* fermented milk; MSFM, mixed-strain fermented milk; NFM, Non-fermented milk; NGS, next-generation sequencing; PCR, polymerase chain reaction; RT-qPCR, reverse-transcription polymerase chain reaction; SCFAs, short-chain fatty acids; qPCR, quantitative polymerase chain reaction.

* Bold denotes primary research outcome.

** Placebo fermented milk prepared without live bacteria.

Table 3 outlines the study design and methods used in animal studies (Uchida and Mogami, 2005; Sprong et al., 2010; Veiga et al., 2010; Lee et al., 2015; Liu et al., 2017; Sevencan et al., 2019; Rabah et al., 2020; Yan et al., 2020; Zhang et al., 2020; Feng et al., 2022; Yang et al., 2022). Test foods included fermented milk (*n =* 5) (Veiga et al., 2010; Lee et al., 2015; Yan et al., 2020; Zhang et al., 2020; Feng et al., 2022), yogurt (*n =* 2) (Liu et al., 2017; Yang et al., 2022), cheese (*n =* 1) (Rabah et al., 2020), cheese whey protein (*n =* 1) (Sprong et al., 2010), milk whey culture (*n =* 1) (Uchida and Mogami, 2005) and kefir (*n =* 1) (Sevencan et al., 2019). In line with the human studies, all test foods included were fermented dairy foods. No study with a non-fermented dairy food (e.g., whole milk) met the study inclusion criteria. Of the fermented milk, three studies investigated mixed-strain fermented milk, one study investigated fermented milk with *L. casei* strains and one study investigated fermented milk with *Bacillus subtilis* strains (Veiga et al., 2010; Lee et al., 2015; Yan et al., 2020; Zhang et al., 2020; Feng et al., 2022). A range of controls were used including water or saline, phosphate-buffered saline (PBS), and acidified or non-fermented dairy among others (Table 3) (Uchida and Mogami, 2005; Sprong et al., 2010; Veiga et al., 2010; Lee et al., 2015; Liu et al., 2017; Sevencan et al., 2019; Rabah et al., 2020; Yan et al., 2020; Zhang et al., 2020; Feng et al., 2022; Yang et al., 2022). Trial duration ranged from 5 days to 4 weeks in length, and test food quantities were provided based on g/kg body weight or measurements ranging from 300uL to 4 mL per day (Uchida and Mogami, 2005; Sprong et al., 2010; Veiga et al., 2010; Lee et al., 2015; Liu et al., 2017; Sevencan et al., 2019; Rabah et al., 2020; Yan et al., 2020; Zhang et al., 2020; Feng et al., 2022; Yang et al., 2022). A range of measures were used to assess gastrointestinal disease status, including histology, ulcer analysis, caecal analysis, colitis score, gut barrier function, and faecal analysis (Uchida and Mogami, 2005; Sprong et al., 2010; Veiga et al., 2010; Lee et al., 2015; Sevencan et al., 2019; Rabah et al., 2020; Yan et al., 2020; Zhang et al., 2020; Feng et al., 2022; Yang et al., 2022). GI symptoms were determined by disease activity analysis, stool analysis (e.g., bleeding, consistency), and intestinal transit time (Sprong et al., 2010; Lee et al., 2015; Liu et al., 2017; Sevencan et al., 2019; Rabah et al., 2020; Yan et al., 2020; Zhang et al., 2020; Feng et al., 2022; Yang et al., 2022). Gut microbiota was assessed using DNA or 16S rRNA sequencing (*n =* 5) (Liu et al., 2017; Yan et al., 2020; Zhang et al., 2020; Feng et al., 2022; Yang et al., 2022) or PCR-based methods (*n =* 3) (Sprong et al., 2010; Veiga et al., 2010; Lee et al., 2015), SCFA concentrations were measured by gas chromatography (*n =* 2) (Veiga et al., 2010; Liu et al., 2017) or UPLC-MS/MS analysis (*n =* 1) (Feng et al., 2022). Most studies (*n =* 9) had several outcomes and did not specify which was their primary outcome (Sprong et al., 2010; Veiga et al., 2010; Lee et al., 2015; Liu et al., 2017; Sevencan et al., 2019; Rabah et al., 2020; Yan et al., 2020; Zhang et al., 2020; Feng et al., 2022). Uchida and Mogami (2005) investigated one outcome, which was gastrointestinal disease status. Yang et al. (2022) investigated several outcomes and stated gut microbial compositional and diversity changes as their primary outcome.

**Table 3.** Methods (animal studies)

Author	Year	Intervention(s)	Quantity (per day)	Control	Duration	Outcome* (method)
Uchida and Mogami (2005)	2005	Milk whey culture	i) 2 g/kg ii) 6 g/kg	Water	9 days	**GID** (histology, ulcer index)
Veiga et al. (2010)	2010	MSFM	100 mg	i) NFM ii) Water	4 weeks	GID (UC score, caecal pH) GM (RT-qPCR) SCFA (GC)
Sprong et al. (2010)	2010	**i)** Cheese whey protein **ii)** Casein **iii)** Casein + Thr/Cys	**i)** 160 g/kg **ii)** 200 g/kg **iii)** 178 g casein +15 g Thr + 7 g Cys	Water	2 weeks	GID (faecal blood loss (HemoQuant)) GIS (diarrhoea assessment) Colonic mucins (fluorometric) GM (qPCR)
Lee et al. (2015)	2015	**i)** *L.cas* BL23 + milk **ii)** *L.cas* BL23 + PBS **iii*)*** *L.cas* BL580 + milk **iv)** *L.cas* BL180 + milk	50uL/d	**i)** PBS **ii)** AM	15 days	GID (histology) GIS (stool consistency, DAI) GM (16S PCR)
Liu et al. (2017)	2017	**i)** Yogurt (2 PB strains) **ii)** Yogurt (3 PB strains)	**i)** 4 mL **ii)** 2 mL **iii)** 1 mL**	**i)** Water **ii)** PB tablets	5 days	GIS (ITT (charcoal transit ratio)) GM (16S sequencing) SCFA (GC)
Sevencan et al. (2019)	2019	Kefir	**i)** 10% kefir (AL) **ii)** 30% kefir (AL)	Water	14 days	GID (macroscopy, histology) GIS (diarrhoea, bleeding assessment)
Rabah et al. (2020)	2020	**i)** Single strain cheese **ii)** Industrial Emmental cheese	400 mg	**i)** PBS **ii)** Sterile control cheese matrix	5 days	GID (histology) GIS (DAI)
Yan et al. (2020)	2020	**i)** MSFM (YS108R) **ii)** MSFM (BB12) **iii)** MSFM (SL)	300uL	NFM	3 weeks	GID (histology, barrier function) GIS (DAI) GM (16S sequencing)
Zhang et al. (2020)	2020	*B. subtilis* FM	300uL	NFM	1 week	GID (histology, barrier function) GIS (DAI) GM (16S sequencing)
Feng et al. (2022)	2022	**i)** PFM **ii)** PPFM	2 mL	Saline	8 days	GID (histology) GIS (DAI) GM (DNA sequencing) SCFAs (UPLC-MS/MS)
Yang et al. (2022)	2022	**i)** LB **ii)** Yogurt **iii)** BT	**i)** 1.2 g/kg **ii)** 0.05 g/kg **iii)** 0.28 g/kg	Saline	10 days	GID (caecal properties) GIS (faecal analysis) **GM** (DNA sequencing)

Abbreviations: AL, ad libitum; AM, acidified milk; *B. subtilis*; *Bacillus subtilis* strain *B. subtilis* JNFE0126; BB12, mixed-strain fermented milk containing *Bifidobacterium animalis* subsp. *lactis* BB12; BT, bifid triple viable capsules; DAI, disease activity index; GC, gas chromatography; GID, gastrointestinal disease; IT, intestinal transit; LB, lacidophilin tablets; *L.cas*, *Lactobacillus casei*; MSFM, mixed-strain fermented milk; NFM, non-fermented milk; PB, probiotic; PBS, phosphate buffer solution; PFM, pasteurised ordinary fermented milk; PPFM, pasteurised probiotic fermented milk (mixed-strain); RT-Thr/Cys, Threonine and Cysteine; SL, mixed-strain fermented milk containing *S.thermophiles* and *Lactobacillus delbrueckii* subsp. *bulgaricus*; qPCR, reverse-transcription polymerase chain reaction; UC, ulcerative colitis; UPLC-MS/MS, ultra-high-performance liquid chromatography-mass spectrometry; YS108R; mixed-strain fermented milk containing *Bifidobacterium longum* YS108R.

* Bold denotes primary research outcome.

** Six intervention arms, two probiotic strain yogurt and three probiotic strain yogurt each administered at 1, 2, and 4 mL per day.

### Gut microbiota and SCFAs

Eight studies with human participants investigated changes in gut microbiota, reporting results as relative bacterial abundance at the order, family, genus, and species levels of the taxonomic hierarchy (Table 4) (Ishikawa et al., 2003; Kato et al., 2004; Matsumoto et al., 2010; Veiga et al., 2014; Liu et al., 2015; Le Nevé et al., 2019; Yilmaz et al., 2019; Li et al., 2020). Gut microbiota alterations were also reported as changes in bacterial alpha diversity (Chao1 index) and bacterial counts by Matsumoto et al. (2010) and *Li et al. (2020*), respectively. These found increases in bacterial alpha diversity and total bacterial counts, relative to baseline measures within experimental groups (Matsumoto et al., 2010; Li et al., 2020). At the genus level, Matsumoto et al. (2010) and Li et al. (2020) saw increases in *Bifidobacterium*, relative to baseline measures within their experimental groups. Both Liu et al. (2015) and Yilmaz et al. (2019) saw increases in *Lactobacillus* at the genus level, relative to control and within experimental group, respectively. Kato et al. (2004) and Veiga et al. (2014) identified increases in several *Bifidobacterium* species (*Bifidobacterium breve, Bifidobacterium pseudocatenulatum, Bifidobacterium animalis),* relative to baseline measures within experimental group and to control, respectively. Six studies investigated SCFA concentrations and reported results as total and/or individual SCFA concentrations (Ishikawa et al., 2003; Kato et al., 2004; Matsumoto et al., 2010; Veiga et al., 2014; Liu et al., 2015; Li et al., 2020). Kato et al. (2004) and Matsumoto et al. (2010) demonstrated increases in total SCFA concentrations within experimental group and relative to control. Most (*n =* 4) of the studies demonstrated increases in butyrate, propionate, and acetate concentrations compared within experimental groups (Matsumoto et al., 2010; Veiga et al., 2014) or relative to controls (Kato et al., 2004; Liu et al., 2015). However, both Ishikawa et al. (2003) and Li et al. (2020) reported decreases in butyrate concentrations, with Li et al. (2020) also reporting decreases in acetate and propionate concentrations, relative to baseline concentrations within experimental groups.

**Table 4.** Gut microbiota and short-chain fatty acid results (human)

Author	Year	*N*	Test food	GID	Change in gut microbiota*	SCFAs
Ishikawa et al. (2003)	2003	21	MSFM	IBD	Species: ↓ *Bifidobacterium vulgatus sp.* ^a^	↓ Butyrate^a^
Kato et al. (2004)	2004	20	MSFM	IBD	Species: ↑ *Bifidobacterium breve, Bifidobacterium pseudocatenulatum* ^a^	↑ Total SCFA^b^ ↑ Butyrate^b^ ↑ Propionate^b^
Matsumoto et al. (2010)	2010	30	LcS FM	FGID	Bacterial counts: ↑ Total bacteria count^a^ Family: ↓ *Enterobacteriaceae* ^a^ Genus: ↑ *Bifidobacterium* ^a^	↑ Total SCFA^a^ ↑ Butyrate^a^ ↑ Propionate^a^ ↑ Acetate^a^
Veiga et al. (2014)	2014	28	FM	IBS	**Species: ↑ *Bifidobacterium animalis, Lactococcus lactis, Streptococcus thermophilus, Lactobacillus subsp. bulgaricus* ^b^** **↓ *Bilophila wadsworthia* ^b^**	↑ Butyrate^a^
Liu et al. (2015)	2015	118	Yogurt	FGID	Genus: ↑ *Lactobacillus* ^b^	↑ Acetate^b^ ↑ Propionate^b^ ↑ Butyrate^b^
Le Nevé et al. (2019)	2019	106	MSFM	IBS	Genus: ↓ *Prevotella/Bacteroides* metabolic potential ratio** ^b^	NR
Yilmaz et al. (2019)	2019	45	Kefir	IBD	Genus: ↑ *Lactobacillus* ^a^	NR
Li et al. (2020)	2020	20	Yogurt	FGID	Alpha diversity (Chao1 index): ↑ Bacterial diversity^a^ Order: ↑ Bacteroidales_unclassified^a^ Family: ↓ Ruminococcaeae_unclassified^a^ Genus: ↑ *Prevotella, Bifidobacterium* ^a^, ↓ *Roseburia, Dialister* ^a^	↓ Acetate^a^ ↓ Propionate^a^ ↓ Butyrate^a^

*Note*: All effects reported are statistically significant (p < 0.05).

Abbreviations: FGID, functional gastrointestinal disorder; FM, fermented milk; GID, gastrointestinal disease; IBD, inflammatory bowel disease; IBD, irritable bowel syndromeLcS FM, *Lactobacillus casei* strain Shirota fermented milk; MSFM, mixed-strain fermented milk; N, number of participants; NR, not reported; SCFAs, short-chain fatty acids.

a Effect within group (comparing pre-intervention and post-intervention).

b Effect between groups (comparing difference between intervention and control groups).

* Bold denotes primary research outcome.

** In high H2 producers only.

A total of eight studies analysed gut microbiota and SCFAs in animal subjects, reporting results as bacterial diversity (alpha) and relative abundance at the phylum, family, genus and species levels (Table 5) (Sprong et al., 2010; Veiga et al., 2010; Lee et al., 2015; Liu et al., 2017; Yan et al., 2020; Zhang et al., 2020; Feng et al., 2022; Yang et al., 2022). The Shannon Index, Richness Index (operational taxonomic unit count), and Chao1 index were used to measure alpha diversity (Liu et al., 2017; Yan et al., 2020; Zhang et al., 2020; Feng et al., 2022). Bacterial alpha diversity consistently increased across four studies, relative to controls (Liu et al., 2017; Yan et al., 2020; Zhang et al., 2020; Feng et al., 2022). At the phylum level, Liu et al. (2017) and Yang et al. (2022) reported increased abundances of Bacteroidetes and decreased abundance of Firmicutes, relative to controls. At the family level, Veiga et al. (2010) and Yan et al. (2020) found that fermented milk decreased *Enterobacteriaceae,* relative to controls. Consistent increases among *Lactobacillus* at the genus level and increases among several *Lactobacillus* species, relative to controls, were identified in four studies (Sprong et al., 2010; Veiga et al., 2010; Zhang et al., 2020; Feng et al., 2022). Fewer animal studies analysed SCFA concentrations compared to human studies, and the results were variable (Table 5). Both Veiga *et al.* and Feng *et al.* saw increases in butyrate in response to fermented milk consumption, whereas Liu *et al.* saw a decrease in butyrate in response to yogurt consumption, relative to controls (Veiga et al., 2010; Liu et al., 2017; Feng et al., 2022). Veiga et al. identified an increase in acetate in response to fermented milk, whereas Liu et al. saw a decrease in acetate in response to yogurt consumption, compared with their respective control groups (Veiga et al., 2010; Liu et al., 2017).

**Table 5.** Gut microbiota and short-chain fatty acid results (animal)

Author	Year	*N*	Test food	Animal, model	Change in gut microbiota (intervention group)*	SCFAs
Veiga et al. (2010)	2010	31	MSFM	Mice, UC	Family: ↓ *Enterobacteriaceae* ^b^ Species: ↑ *Bifidobacterium lactis, Streptococcus thermophilus, Lactobacillus subsp. bulgaricus, Lactococcus lactis* ^b^	↑ Acetate^b^ ↑ Propionate^b^ ↑ Butyrate^b^ ↓ Lactate^b^
Sprong et al. (2010)	2010	48	i) CWP ii) Casein iii) Casein + Thr/Cys	Rats, UC	Genus: ↑ *Bifidobacterium, Lactobacillus* (CWP and Thr/Cys)^b^	NR
Lee et al. (2015)	2015	48	i) *L.cas* BL23 + milk ii) *L.cas* BL580 + milk iii) *L.cas* BL180 + milk	Mice, UC	Family: ↑*Commondacea, Bifidobacteriaceae* (BL32) ↓ *Clostridiaceae* (BL580)^b^	NR
Liu et al. (2017)	2017	144	i) Yogurt (2 PB strains) ii) Yogurt (3 PB strains)	Mice, FC	Alpha diversity (bacterial richness (OTU)): ↑ Bacterial richness (both groups)^b^ Phylum: ↑ Bacteroidetes (both groups)^b^ ↓ Firmicutes (both groups)^b^	↓ Acetate (Y2)^b^ ↓ Butyrate (Y3)^b^
Yan et al. (2020)	2020	40	i) MSFM (YS108R) ii) MSFM (BB12) iii) MSFM (SL)	Mice, UC	Alpha diversity (Shannon index): ↑ Diversity (YS108R, BB12)^b^ Phylum: ↓ Proteobacteria (all groups)^b^ Family: ↓ *Enterobacteriaceae* (all groups)^b^ ↑ *Lachnospiraceae* (BB12, YS108R)^b^	NR
Zhang et al. (2020)	2020	100	*B. subtilis* FM	Mice, IBD	Alpha diversity (Shannon & Chao1 Index): ↑ Diversity^b^ Genus: ↑*Bacillus, Alloprevotella*, *Ruminococcus* ↑ *Alistipes*, *Lactobacillus* ^b^ Family: ↓ *Lachnospiraceae, Bacteroidaceae* ↑ *Lactobacillaceae* ^b^	NR
Feng et al. (2022)	2022	32	i) FM ii) PFM	Rats, IBD	Alpha Diversity (Richness Index): ↑ Diversity (PFM)^b^ Species: ↓ *Alistipes shahii, Muribaculaceae, Alistipes obesi.* ↑ *Akkermansia muciniphila, Dorea sp. CAG:317, Clostridium sp. CAG:306, Azospirillum, Enterococcus faecalis, Bacteroides oleicplenus, Bacteroides acidifaciens* (PFM)^b^ Species: ↑ *Lactobacillus animalis, Lactobacillus johnsonii, Bacteroides intestinalis, Bacteroides thetaiotaomicron, Parabacteroides merdae* (both groups)^b^	↑ Butyrate (PFM)^b^ ↑ Succinate (PFM)^b^ ↑ Benzoate (PFM)^b^
Yang et al. (2022)	2022	40	Yogurt	Mice, AAD	**Phylum: Restoration of Firmicutes and Bacteroidetes to normal levels^b^** **↓ Proteobacteria^b^** **Family: ↓ *Bacteroidaceae* ^b^** **Genus: ↓ *Bacteroides*** **↓ *Parasutterella* ^b^**	NR

*Note*: All effects reported are statistically significant (p < 0.05).

* Bold denotes primary research outcome.

a Effect within group (comparing pre-intervention and post-intervention).

b Effect between groups (comparing difference between intervention and control groups).

Abbreviations: AAD, antibiotic-associated diarrhoea; BB12, mixed-strain fermented milk containing *Bifidobacterium animalis* subsp. *lactis* BB12; *B. subtilis*; *Bacillus subtilis* strain *B. subtilis* JNFE0126; CWP, cheese whey protein; FC, functional constipation; FM, fermented milk; IBD, inflammatory bowel disease; *L.cas*, *Lactobacillus casei*; MSFM, mixed-strain fermented milk; N, number of participants; NR, not reported; OTU, operational taxonomic units; PB, probiotic; PFM, probiotic fermented milk; SL, mixed-strain fermented milk containing *S.thermophiles* and *Lactobacillus delbrueckii* subsp. *bulgaricus*; Thr/Cys, Threonine and Cysteine; UC, ulcerative colitis; Y2, yogurt with two probiotic strains; Y3 yogurt with three probiotic strains; YS108R; mixed-strain fermented milk containing *Bifidobacterium longum* subsp. *longum* YS108R.

### Gastrointestinal health

A total of 14 studies investigated gastrointestinal symptoms and disease status response to dairy consumption in humans (Table 6) (Beniwal et al., 2003; Ishikawa et al., 2003; Kato et al., 2004; Matsumoto et al., 2010; Søndergaard et al., 2011; Thijssen et al., 2011; Marteau et al., 2013; Tilley et al., 2014; Gomi et al., 2015; Liu et al., 2015; Le Nevé et al., 2019; Yilmaz et al., 2019; Li et al., 2020; Mokhtar et al., 2021). Overall, improvements in gastrointestinal health, individual symptoms (e.g., bloating, flatulence), and defecation parameters in response to fermented milk, kefir, or yogurt consumption were reported (Beniwal et al., 2003; Ishikawa et al., 2003; Kato et al., 2004; Matsumoto et al., 2010; Søndergaard et al., 2011; Thijssen et al., 2011; Marteau et al., 2013; Tilley et al., 2014; Gomi et al., 2015; Liu et al., 2015; Le Nevé et al., 2019; Yilmaz et al., 2019; Li et al., 2020; Mokhtar et al., 2021). Five studies found that fermented milk and yogurt intakes regulated defecation frequency, comparing intervention groups at baseline and post-intervention (Matsumoto et al., 2010; Liu et al., 2015; Li et al., 2020; Mokhtar et al., 2021), whereas Beniwal *et al.* reported effects relative to control (Beniwal et al., 2003). Three studies found that fermented milk and yogurt consumption improved stool consistency, comparing baseline and post-intervention measures within intervention groups (Matsumoto et al., 2010; Liu et al., 2015), or relative to control (Tilley et al., 2014). Improvements in gastrointestinal symptoms and gut comfort were reported across five studies (Søndergaard et al., 2011; Thijssen et al., 2011; Le Nevé et al., 2019; Yilmaz et al., 2019; Mokhtar et al., 2021). Within these, Mokhtar et al. and Søndergaard et al. found that fermented milk improved gastrointestinal symptoms, comparing baseline and post-intervention symptoms within intervention groups (Søndergaard et al., 2011; Mokhtar et al., 2021). Improved gut comfort in response to fermented milk consumption was demonstrated, relative to control, by Le Néve et al., and within intervention group by Thijssen et al. (Thijssen et al., 2011; Le Nevé et al., 2019). Yilmaz et al. found kefir consumption improved bloating, relative to control (Yilmaz et al., 2019). Kato et al. and Gomi et al. saw improvements in self-reported disease status among UC and FGID patients, respectively, in response to fermented milk intake (Kato et al., 2004; Gomi et al., 2015). These effects were shown by comparing disease status between intervention and control groups by Kato et al., and within intervention group by Gomi et al. (Kato et al., 2004; Gomi et al., 2015). Additionally, Kato et al. saw significantly lower endoscopic activity index and histological scores from baseline to post-intervention within the experimental group (Kato et al., 2004). No study reported a deterioration in gastrointestinal disease status or symptoms in response to dairy consumption.

**Table 6.** Gastrointestinal disease status and symptoms (human)

Author	Year	N	Test food	GID	GI symptoms and disease status*
Ishikawa et al. (2003)	2003	21	MSFM	UC	**Exacerbation of disease in control group relative to BFM group^b^**
Beniwal et al. (2003)	2003	202	Yogurt	AAD	**Reduced diarrhoea frequency^b^**
Kato et al. (2004)	2004	20	MSFM	UC	**Lower clinical activity index^b^** **Lower endoscopic activity index and histological score^a^**
Matsumoto et al. (2010)	2010	30	LcS FM	FD	Decreased defecation frequency^a^ Improved stool consistency^a^
Søndergaard et al. (2011)	2011	52	i) FM ii) AM	IBS	**Increased symptom relief (both groups)^a^**
Marteau et al. (2013)	2013	530	MSFM	FGID	**Improved in GI well-being^b^**
Tilley et al. (2014)	2014	106	LcS FM	FGID	**Improved stool consistency^b^**
Gomi et al. (2015)	2015	27	MSFM	FGID	**Decreased gastric symptom score^a^**
Liu et al. (2015)	2015	118	Yogurt	FC	**Decreased stool hardness and incomplete evacuation sensations^a^** **Increased defecation frequency^a^**
Thijssen et al. (2011)	2016	80	LcS FM	IBS	Improved discomfort, flatulence scores** ^a^
Le Nevé et al. (2019)	2019	106	MSFM	IBS	Decreased GI discomfort*** ^b^
Yilmaz et al. (2019)	2019	45	Kefir	IBD	Decreased bloating scores^b^ Increased ‘feeling good’ scores^b^
Li et al. (2020)	2020	20	Yogurt	FC	Increased defecation frequency^a^
Mokhtar et al. (2021)	2021	165	FM	IBS-C	Improved symptoms^a^ Reduced ITT^a^

*Note*: All effects reported are statistically significant (p < 0.05).

* Bold denotes primary research outcome.

** At long-term follow-up only.

*** Groups stratified by H2 exhalation levels (high vs low) with reported effect identified in high H2 group only.

a Effect within group (comparing pre-intervention and post-intervention).

b Effect between groups (comparing difference between intervention and control groups).

Abbreviations: AAD, antibiotic-associated diarrhoea; AM, acidified milk; FD, functional diarrhoea; FGID, functional gastrointestinal disorder; FM, fermented milk; GID, gastrointestinal disease; GI, gastrointestinal; IBD, irritable bowel syndrome; IBS-C, IBS with constipation; ITT, intestinal transit time; LcS FM, *Lactobacillus casei* strain Shirota fermented milk; MSFM, mixed-strain fermented milk; N, number of participants; NR, not reported; UC, ulcerative colitis.

Ten studies analysed gastrointestinal symptoms and disease status in response to dairy intake in animal cohorts (Table 7) (Uchida and Mogami, 2005; Sprong et al., 2010; Lee et al., 2015; Liu et al., 2017; Sevencan et al., 2019; Rabah et al., 2020; Yan et al., 2020; Zhang et al., 2020; Feng et al., 2022; Yang et al., 2022). Four studies identified a reduction in disease activity index in response to dairy in the form of cheese (Rabah et al., 2020) or fermented milk (Yan et al., 2020; Zhang et al., 2020; Feng et al., 2022), relative to controls. Mucosal healing and reduction in colonic damage in response to fermented milk consumption were demonstrated in four studies, relative to controls (Yan et al., 2020; Zhang et al., 2020; Feng et al., 2022) and within the intervention group (Uchida and Mogami, 2005). Sevencan et al. saw a decreased colonic weight/length ratio in response to kefir intake (Sevencan et al., 2019). Yan et al. and Sprong et al. saw increased MUC2 expression and increased faecal mucin excretion in response to fermented milk and cheese whey protein, respectively, relative to controls (Sprong et al., 2010; Yan et al., 2020). Four studies overall saw reduced diarrhoea prevalence in response to fermented dairy intake (Sprong et al., 2010; Lee et al., 2015; Sevencan et al., 2019; Yang et al., 2022). Within these, three studies saw a reduction in diarrhoea relative to controls for fermented milk (Lee et al., 2015), kefir (Sevencan et al., 2019), and yogurt intakes (Yang et al., 2022). Sprong et al. saw that cheese whey protein reduced diarrhoea, comparing changes from baseline to post-intervention within the intervention group (Sprong et al., 2010). Sprong et al. and Lee et al. found cheese whey protein and fermented milk reduced faecal blood loss and rectal bleeding, respectively, relative to controls (Sprong et al., 2010; Lee et al., 2015). Additional findings for individual studies are reported in Table 7.

**Table 7.** Gastrointestinal disease status and symptoms (animal)

Author	Year	*N*	Animal, model	Test food	GI symptoms	Clinical	Endoscopy and colonoscopy*
Uchida and Mogami (2005)	2005	NR	Rats, UC	Milk whey culture	NR	NR	**Reduced ulcer index^b^** **Colonic musical healing (epithelial regeneration)^a^**
Sprong et al. (2010)	2010	48	Rats, UC	i) CWP ii) Casein iii) Casein + Thr/Cys	Reduced diarrhoea (CWP and Casein + Thr/Cys groups)^a^	Lowered faecal blood loss (Casein + Thr/Cys)^b^ Increased mucin excretion in (CWP, Casein + Thr/Cys)^b^	NR
Lee et al. (2015)	2015	48	Mice, UC	*L.cas* BL23 + milk	Reduced diarrhoea^b^	Reduced rectal bleeding^b^	NR
Liu et al. (2017)	2017	144	Mice, FC	i) Yogurt (2 PB) ii) Yogurt (3 PB)	NR	Increased ITT in (3 PB)^b^	NR
Sevencan et al. (2019)	2019	54	Rats, UC	Kefir ()	Reduced diarrhoea (kefir10%)^b^	NR	Lower colonic weight/length ratio (kefir10%)^b^
Yan et al. (2020)	2020	40	Mice, UC	i) MSFM (YS108R) ii) MSFM (BB12) iii) MSFM (SL)	NR	Maintained tight junction proteins and increased MUC2 expression (YS108R)^b^ Decreased DAI (YS108R)^b^	Prevented mucosal layer damage (YS108R)^b^
Zhang et al. (2020)	2020	100	Mice, IBD	*B. subtilis* FM	NR	Decreased DAI^b^	Intestinal mucosal injury attenuated^b^
Rabah et al. (2020)	2020	90	Mice, UC	i) Single strain cheese ii) Industrial Emmental cheese	NR	Decreased DAI (both cheese groups)^b^	Significant reduction in histopathological score (Emmental group)^b^
Feng et al. (2022)	2022	32	Rats, IBD	i) FM ii) PFM	NR	Decreased DAI (PFM)^b^	Alleviated colonic damage (PFM)^b^
Yang et al. (2022)	2022	40	Mice, AAD	Yogurt	Decreased diarrhoea scores^b^	NR	Inhibited increased cecum length and caecal index^b^

*Note*: All effects reported are statistically significant (p < 0.05).

* Bold denotes primary research outcome.

a Effect within group (comparing pre-intervention and post-intervention).

b Effect between groups (comparing difference between intervention and control groups).

Abbreviations: AAD, antibiotic-associated diarrhoea; BT, bifid triple viable capsules; BB12, mixed-strain fermented milk containing *Bifidobacterium animalis* subsp. *lactis* BB12; CWP, Cheese whey protein; DAI, disease activity index; FC, functional constipation; FM, fermented milk; GI, gastrointestinal; IBD, inflammatory bowel disease; ITT, intestinal transit time; LB, lacidophilin tablets; MSFM, mixed-strain fermented milk; N, number of participants; NR, not reported; PBS, phosphate buffered saline; PB, probiotic strains; PFM; Probiotic fermented milk; SL, mixed-strain fermented milk containing *S.thermophiles* and *Lactobacillus delbrueckii* subsp. *bulgaricus*; *B. subtilis*; *Bacillus subtilis* strain *B. subtilis* JNFE0126; Thr/Cys, Threonine and Cysteine; YS108R; mixed-strain fermented milk containing *Bifidobacterium longum* YS108R; UC, ulcerative colitis.

### Risk of bias

Risk of bias in most studies with human participants was rated as ‘some concerns’ (*n =* 13) (Beniwal et al., 2003; Ishikawa et al., 2003; Kato et al., 2004; Matsumoto et al., 2010; Søndergaard et al., 2011; Thijssen et al., 2011; Marteau et al., 2013; Veiga et al., 2014; Gomi et al., 2015; Liu et al., 2015; Yilmaz et al., 2019; Li et al., 2020; Mokhtar et al., 2021). The main sources of potential bias were deviations from intended interventions, measurement of the outcome, and selection of the reported result (Figure S1). Missing information required for thorough bias assessment also influenced these results. Tilley *et al.* and Le Neve *et al.* were considered to have low risk of bias in their study designs (Tilley et al., 2014; Le Nevé et al., 2019). Risk of bias in studies with animal participants were mostly rated as ‘some concerns’ (*n =* 9) (Uchida and Mogami, 2005; Sprong et al., 2010; Veiga et al., 2010; Lee et al., 2015; Sevencan et al., 2019; Yan et al., 2020; Zhang et al., 2020; Feng et al., 2022; Yang et al., 2022), whereas Liu et al. and Rabah et al. were rated as ‘low with some concerns’ (Liu et al., 2017; Rabah et al., 2020). The main sources of potential bias across the studies were within the allocation concealment, random housing, and blinding domains. This was primarily due to a lack of information provided on these study design parameters.

The scope of this review focused on significant findings and has not reported on findings where no change was identified, or where a non-significant change was identified. We recognise this is important and the data extraction file which includes non-significant and ‘no change’ findings, where reported, is provided in the Supplementary material.

## Discussion

Considering the evidence presented in this review, it appears that overall, fermented dairy foods can positively influence aspects of gastrointestinal health and the gut microbiome in IBD and FGID cohorts. Gastrointestinal bacterial alpha diversity consistently increased in response to fermented dairy consumption in both human and animal studies (Matsumoto et al., 2010; Liu et al., 2017; Li et al., 2020; Yan et al., 2020; Zhang et al., 2020; Feng et al., 2022). Gut microbial abundances can be reported at several levels within bacterial taxonomy (from phylum to sub-species levels), introducing limitations when comparing studies reporting results at different levels within the taxonomic hierarchy (Hugenholtz et al., 2021). However, a strong trend of increased relative *Lactobacillus* and *Bifidobacterium* abundances, and certain species within these genera, emerged (Kato et al., 2004; Matsumoto et al., 2010; Sprong et al., 2010; Veiga et al., 2010, 2014; Liu et al., 2015; Yilmaz et al., 2019; Li et al., 2020; Zhang et al., 2020). This was shown in studies using a range of fermented dairy test foods (fermented milks, kefir, yogurt, and cheese whey protein), providing supporting evidence that fermented dairy foods can positively influence gut microbial characteristics (Kato et al., 2004; Matsumoto et al., 2010; Sprong et al., 2010; Veiga et al., 2010, 2014; Liu et al., 2015; Yilmaz et al., 2019; Li et al., 2020; Zhang et al., 2020). *Lactobacillus* and *Bifidobacterium* are considered commensal gut genera, wherein increased relative abundances have been shown to benefit the host (O’Callaghan and van Sinderen, 2016; Hidalgo-Cantabrana et al., 2017; Dempsey and Corr, 2022; Rastogi and Singh, 2022). Thus, increasing the intake of fermented dairy foods may ultimately provide part of a solution in correcting apparent gut microbial dysbiosis in such gastrointestinal disease cohorts.

SCFAs are produced by gut microbes through colonic fermentation of fibre, and resistant starches, and certain SCFAs help to maintain gut and immune homeostasis (Tan et al., 2014). Butyrate, propionate, and acetate are beneficial SCFAs, and faecal concentrations of these SCFAs are reduced in gastrointestinal disease cohorts (Parada Venegas et al., 2019). Pooling human and animal data, most studies (*n =* 6) showed increases in total SCFAs, butyrate, propionate, and acetate in response to fermented dairy (Kato et al., 2004; Matsumoto et al., 2010; Veiga et al., 2010; Veiga et al., 2014; Liu et al., 2015; Feng et al., 2022). However, in contrast to this, three studies reported decreases in butyrate, two reported decreases in acetate and one study showed a decrease in propionate concentrations (Ishikawa et al., 2003; Liu et al., 2017; Li et al., 2020). Considering studies reporting findings relative to controls only, it is worth noting that four studies reported increases across SCFA concentrations (Kato et al., 2004; Veiga et al., 2010; Liu et al., 2015; Feng et al., 2022), whereas just one study reported a decrease (Liu et al., 2017). Therefore, considering these studies only (which are more statistically robust), most studies (4 out of 5) showed fermented dairy intakes improved faecal SCFA profiles (Kato et al., 2004; Veiga et al., 2010; Bland and Altman, 2011; Liu et al., 2015; Liu et al., 2017; Feng et al., 2022). In addition, interpreting faecal SCFA concentrations in isolation is difficult, without considering fibre and resistant starch intakes, as gut microbes require these substrates to produce SCFAs (Tan et al., 2014). Therefore, dairy consumption alone cannot directly influence SCFA concentrations without fibre and resistant starch present in the colon, thus, this may explain some of the variability across findings for this outcome. It is also worth noting the heterogeneity across different methods used to analyse SCFAs (e.g., HPLC, gas chromatography, UPLC-MS/MS, *in vitro* analysis), which may also explain some of the variability in the results.

In human studies, gastrointestinal health parameters were primarily assessed through self-reported measures, wherein a strong trend of improved symptoms in response to fermented dairy consumption emerged (Beniwal et al., 2003; Ishikawa et al., 2003; Kato et al., 2004; Matsumoto et al., 2010; Søndergaard et al., 2011; Thijssen et al., 2011; Marteau et al., 2013; Tilley et al., 2014; Gomi et al., 2015; Liu et al., 2015; Le Nevé et al., 2019; Yilmaz et al., 2019; Li et al., 2020; Mokhtar et al., 2021). Most notably, defecation parameters (including defecation frequency, stool consistency and intestinal transit time) were consistently improved (Beniwal et al., 2003; Matsumoto et al., 2010; Tilley et al., 2014; Liu et al., 2015; Li et al., 2020; Mokhtar et al., 2021). In agreement with this, animal models also demonstrated improved defecation parameters in response to fermented dairy intake, based on faecal analysis methods (Sprong et al., 2010; Lee et al., 2015; Sevencan et al., 2019; Yang et al., 2022). Gastrointestinal disorders significantly affect the quality of life, and patients experience considerable discomfort and distress associated with their symptoms (Hahn et al., 1999). Based on these findings, fermented dairy consumption may be a useful tool to alleviate some of the gastrointestinal discomfort experienced by IBD and FGID patients. While animal studies cannot capture self-reported gastrointestinal parameters, they do facilitate more invasive measurements of gastrointestinal health, such as colonic histological analysis. Colonic histology allows in-depth analysis of the colonic environment, and is particularly important in relation to IBD in clinical practice (Kellermann and Riis, 2021). In the animal studies presented, dairy interventions improved clinical gastrointestinal parameters, with notable improvements in colonic mucosal healing and reduced colonic damage, measured via colonic histology (Uchida and Mogami, 2005; Sevencan et al., 2019; Yan et al., 2020; Zhang et al., 2020; Feng et al., 2022). In line with these findings, one human study showed lower endoscopic activity index and histological score in response to fermented milk intake (Søndergaard et al., 2011). Compiling mostly self-reported findings in human cohorts with colonic histological findings in animal cohorts, it appears that fermented dairy can improve a range of gastrointestinal health parameters in IBD and FGID patients.

The improvement in gastrointestinal symptom parameters seen in humans may be attributed to the mucosal healing and reduction in colonic damage demonstrated in comparable animal studies, but this association requires further research. Future human studies should investigate gastrointestinal health status via non-subjective methods. Examples of this may include gut barrier function analysis and colonic histology analysis (Grootjans et al., 2010; Rosenberg et al., 2013; Liu et al., 2019). Intestinal barrier function can be assessed by non-invasive methods, e.g., serum intestinal fatty acid binding protein concentration (Grootjans et al., 2010). Although performing colonic biopsies is invasive, IBD patients undergo routine colonoscopies wherein biopsies are taken (Kellermann and Riis, 2021). Thus, there is an opportunity to further explore this area through conducting colonic histological analysis in humans while adhering to ethics in clinical research settings, as demonstrated in other studies (Rosenberg et al., 2013; Liu et al., 2019). This type of analysis would add to the body of human evidence in this area, which currently relies mostly on self-reported gastrointestinal health measures, which are subjective, and have potential inherent bias (Althubaiti, 2016).

While this review highlights improvements in gastrointestinal health in response to fermented dairy, there are several limitations and points to consider when interpreting the results. Study design parameters including test food types and their quantities, controls, analysis methods and reporting of results were widely variable across studies. This review pools evidence from the studies, irrespective of this heterogeneity, therefore, these findings should be interpreted with caution. Dairy test foods included in this review are largely variable, in terms of their physical structures (e.g., yogurt is gel/viscoelastic, milk is liquid) and their nutritional profiles (e.g., proteins content, whey/casein ratio, fat content, fat structure) (Thorning et al., 2017). As noted by Thorning et al., these aspects of variability across dairy foods can influence the biological responses associated with consumption (Thorning et al., 2017). For the purpose of this review, we analysed dairy foods as a whole, without delving into the apparent variability due to physical structures and nutritional matrices within and between the dairy foods. Future work in this area is needed exploring the role of dairy food matrix variables. In addition, there was large variability in outcome reporting methods within and between studies. Studies reported findings as differences within experimental groups (baseline vs post-intervention), or as differences between experimental and control groups. Reporting findings relative to control provides more statistically robust evidence, and future studies should aim to report results in this way (Bland and Altman, 2011). Lastly, as noted in the results, half of the studies overall (*n =* 13) investigated several outcomes without specifying a primary research outcome, and several of the findings reported across the studies were secondary outcomes. Primary and secondary outcome findings were included in the data synthesis with equal importance, so this should be considered when interpreting the results. While this review provides a comprehensive overview of the research to date, it is important to note the significant heterogeneity across study design parameters, the study quality and validity of results reported.

Another limitation is the lack of nutritional information provided for test foods. Only three studies provided detailed nutritional information for test foods (Supplementary Table S3) (Matsumoto et al., 2010; Tilley et al., 2014; Liu et al., 2015). When foods are digested, their nutritional components (e.g., macronutrients, polyphenols, probiotics) and endogenous metabolites interact with the gastrointestinal environment, wherein food nutritional properties can influence the gut microbiome and the gastrointestinal environment in different ways (Zhang, 2022). Due to the lack of information available, it was not feasible to delve into the nutritional properties of test foods across different studies, to further understand their impact on gut microbiota and gastrointestinal health. Therefore, future studies should include comprehensive nutritional information of test foods to allow deeper understanding of how dairy nutritional components can influence gastrointestinal parameters. Further, very few human studies considered dietary intake as a potential cofounder in their analysis of changes in gut microbial characteristics or gastrointestinal health. Animal studies allow strict control over dietary intake (nutritional intake beyond test foods), and monitoring and controlling for this in human gastrointestinal research is a major challenge (Staudacher et al., 2022). In line with specific nutritional components within test foods, overall dietary intake (beyond test foods) is a strong predictor of gut microbial composition and gastrointestinal health, and should be considered and controlled for accordingly (Hasan and Yang, 2019; Staudacher et al., 2022). While most studies instructed that participants maintained their habitual diet and refrained from dairy, fermented dairy and/or probiotics, just one study out of the 15 human studies assessed dietary intake and considered it as a potential cofounder in their analysis (monitored macronutrient, micronutrient and fibre intakes at baseline and post-intervention) (Marteau et al., 2013). Further information on controlling for dietary intake as a potential cofounder can be found in the supplementary material (data extraction form). Although it is challenging to account for dietary intake variability in free-living human cohorts, future studies should consider assessing dietary intake and specific relevant dietary components (e.g., fibre intake) in their analysis of gut microbiota alterations and changes in gastrointestinal parameters in dietary intervention studies.

In relation to test foods, although this review aimed to explore dairy foods, including both fermented and non-fermented, all test foods included in the data synthesis have a fermented aspect. Therefore, many of the test foods contained probiotics (e.g., fermented milk with probiotics). This considered, it could be argued that the positive gastrointestinal effects shown for these foods are influenced by the probiotics (e.g., *Bifidobacterium* strains in fermented milk), rather than the dairy foods themselves. However, while evidence shows that probiotic bacteria exert positive gastrointestinal effects, it is also important to consider the probiotic delivery matrix (Parker et al., 2018). As shown by Liu et al., administering identical probiotic strains in different matrices (yogurt versus tablet) elicited contrasting effects, wherein gastrointestinal improvements were observed only in the yogurt group (Liu et al., 2017). Similarly, Lee et al. also showed the benefits of *L.cas* BL23 were dependent on the delivery matrix, wherein significant benefits were only shown in the dairy delivery matrix (milk), compared with administration in PBS (Lee et al., 2015). This suggests an additive effect of the matrix in addition to the probiotic content. Further, beyond these studies, sufficient evidence shows that dairy foods, particularly milk and yogurt, are excellent matrices for probiotic delivery, in relation to preserving probiotic viability (Morelli et al., 2018; Rodrigues et al., 2019). Although most test foods in this review include probiotics, the dairy delivery matrix is an additional consideration that warrants further investigation. Just two studies in this review explored the role of the dairy matrix in probiotic administration, therefore, for the majority of studies presented here, it is difficult to differentiate the effects of the dairy matrix from the probiotics themselves.

Additionally, the studies presented here highlight the effects of a range of fermented dairy food types containing probiotics on gastrointestinal health. Different dairy foods (e.g., yogurt, fermented milk, cheese) have heterogenous structural and nutritional properties, and previous studies show that the dairy matrix plays a role in the biological response to their consumption (Thorning et al., 2017; Aguilera, 2019). Thus, comparing the matrix effect across different dairy food types (e.g., fermented milk, yogurt) with respect to probiotic delivery also warrants further investigation, with respect to gastrointestinal health in IBD and FGID cohorts. There is opportunity to examine the effects of probiotics administered in dairy foods vs control, and then to also compare the dairy delivery matrix across different dairy foods.

While fermented foods and their nutritional compounds are shown to exert positive effects on gut microbial characteristics, it should be noted that current technologies may not be sensitive enough to detect small microbiota alterations (Mousa et al., 2022; Ng et al., 2023). This considered, although foods may not significantly alter gut microbial characteristics, they can still confer benefits to the host through metabolites produced or through interaction with the host’s immune system, which are difficult to capture (Ng et al., 2023). Further advancements in gut microbiome analysis methods will allow a deeper understanding of the effects of fermented dairy foods on the gut microbial ecosystem, beyond the scope of relative bacterial abundance and diversity (Mousa et al., 2022). In addition to this, assessing changes in gut microbial composition in conjunction with changes in gastrointestinal health (e.g., symptoms) is also important to capture the effects of fermented dairy foods on the gut microbiota, and the subsequent gastrointestinal health benefits which may be associated with gut microbial alterations.

There are also opportunities for future research to explore a wider range of dairy food types. Test foods in human studies were restricted to fermented milks, kefir, and yogurt only, whereas animal studies explored a wider range of test foods providing promising results. Notably, cheese and cheese whey protein both increased relative abundances of *Bifidobacterium* and *Lactobacillus* while also improving clinical gastrointestinal parameters (Sprong et al., 2010; Rabah et al., 2020). These findings provide a rationale to explore a wider range of dairy foods in this context in humans. Alongside yogurt, cheese is the most commonly consumed form of fermented dairy (González et al., 2019). Thus, from a practical perspective, cheese is an important food to consider moving forward in the exploration of fermented dairy on the gut microbiome and gastrointestinal health. In addition, a deeper understanding of how fermented dairy foods influence the gut microbiome and gastrointestinal health is needed. The specific food components and the mechanisms in which they influence beneficial changes in the gut microbiome and gut symptoms warrants further investigation. Future work should expand test foods, while also considering the dairy food components influencing gastrointestinal effects, and the mechanisms by which they act.

To conclude, this review provides a basis of evidence showing fermented bovine dairy foods can improve gut microbial dysbiosis and gastrointestinal parameters in IBD and FGID cohorts. IBD and FGIDs severely affect quality of life, and while symptoms can be managed through clinical and dietary strategies, there is no cure (Zuo and Ng, 2018). Thus, dietary management is highly important in such cohorts. Increasing fermented dairy consumption is a practical dietary strategy that may aid the management of gastrointestinal complications. However, further well-designed large-scale human studies considering both clinical and self-reported gastrointestinal health measures and explore a wider range of test foods are now needed to extend and strengthen the existing evidence. It is worth noting that the only European Food Safety Authority approved health claim associated with fermented dairy is in relation to yogurt: ‘live yogurt cultures can improve digestion of yogurt lactose in individuals with lactose maldigestion’ (EFSA Panel on Dietetic Products N, Allergies, 2010). Future studies in this area may inform potential health claims associated with fermented dairy foods and gastrointestinal health, in relation to the gut microbiome and gastrointestinal symptoms.

## Supporting information

Ní Chonnacháin et al. supplementary material 1 Ní Chonnacháin et al. supplementary material

Ní Chonnacháin et al. supplementary material 2 Ní Chonnacháin et al. supplementary material

## Supplementary material

The supplementary material for this article can be found at http://doi.org/10.1017/gmb.2024.2.

## Research transparency and reproducibility

The data extraction form, which includes extensive study details of the papers included in this review, is provided as Supplementary material and can be made publicly available.
