# Supplementary material for: The effects of dairy on the gut microbiome and symptoms in gastrointestinal disease cohorts: a systematic review
Source: Gut Microbiome (Camb). 2024 May 7;5:e5. doi: 10.1017/gmb.2024.2 (PMC11406376; doi:10.1017/gmb.2024.2)
Supplement: Ní Chonnacháin et al. supplementary material 2 — Ní Chonnacháin et al. supplementary material [file S2632289724000021sup002.docx]

**Supplementary material**

**Extended search strategy**

**Database 1:** PubMed

“dairy product*”[tw] OR milk[tw] OR “bovine milk”[tw] OR “cow milk*”[tw] OR “milk product*”[tw] OR  cheese[tw] OR butter[tw] OR cream[tw] OR “ice cream”[tw] OR “fermented milk”[tw] OR yogurt[tw] OR yoghurt[tw] OR kefir[tw] OR casein[tw] OR whey[tw] OR “casein hydrolysate”[tw] OR beta-casein[tw] OR “whey hydrolysate”[tw] OR “fermented whey”[tw] OR “fermented dairy”[tw] OR “Dairy Products”[Mesh] OR “Cultured Milk Products”[Mesh] OR “Caseins”[Mesh] OR “Whey”[Mesh] OR “Whey proteins”[Mesh] AND “gut microb*”[tw] OR “gut microbiota”[tw] OR “intestinal microbiota”[tw] OR “intestinal microbiome”[tw] OR “bacterial abundance”[tw] OR “bacterial richness”[tw] OR “bacterial count*”[tw] OR “bacterial diversity”[tw] OR “gut microbial diversity”[tw] OR “intestinal microbial diversity”[tw] OR “short-chain fatty acid*”[tw] OR “SCFA”[tw] OR “Gastrointestinal Microbiome”[Mesh] OR “Fatty Acids, Volatile”[Mesh] AND **“**gut integrity”[tw] OR “intestinal integrity”[tw] OR “gut permeability”[tw] OR “intestinal permeability”[tw] OR “intestinal barrier function”[tw] OR “gut barrier function”[tw] OR “tight junction*”[tw] OR “bowel motion*”[tw] OR “bowel frequency”[tw] OR “stool frequency”[tw] OR “stool consistency”[tw] OR “abdominal distension”[tw] OR “intestinal gas”[tw] OR constipation[tw] OR “abdominal pain”[tw] OR “gastrointestinal discomfort”[tw] OR “FGID”[tw] OR “IBS”[tw] OR “Gastrointestinal Disease*”[Mesh] OR “Inflammatory Bowel Diseases”[Mesh] OR “Digestive System Diseases”[Mesh] OR “Irritable Bowel Syndrome”[Mesh] OR “Diarrhea”[Mesh] OR “Constipation”[Mesh] OR “Abdominal pain”[Mesh] OR “Gastrointestinal Transit”[Mesh] OR “Gastrointestinal Motility”[Mesh]

**Database 2:** Embase

**Concept 1:** 'dairy product'/exp OR 'fermented dairy product'/exp OR 'cow milk'/exp OR 'casein'/exp OR 'casein hydrolysate'/exp OR 'fermented milk drink'/exp

**Concept 2:** 'intestine flora'/exp OR 'gut microbiome'/exp OR 'bacterial abundance'/exp OR 'bacterial richness'/exp OR 'bacterial diversity'/exp OR 'short chain fatty acid'/ex

**Concept 3 :** 'gut permeability'/exp OR 'intestinal barrier'/exp OR 'tight junction'/exp OR 'digestive function'/exp OR 'gastrointestinal tract function'/exp OR 'digestive system disease'/exp OR 'constipation'/exp OR 'digestive system function disorder'/exp OR 'inflammatory bowel disease'/exp OR 'gut integrity' OR 'bowel motion' OR 'stool frequency' OR 'abdominal distention' OR 'gastrointestinal comfort' OR 'gastrointestinal motility'

Combined #1 AND #2 AND #3 for final search

**Database 3:** Web of Science

dairy OR dairy product* OR fermented dairy product OR milk OR yogurt OR yoghurt OR cheese OR cream OR butter OR kefir OR cultured milk OR cow milk OR bovine milk OR casein OR casein hydrolysate OR whey OR whey hydrolysate OR fermented milk drink OR fermented dairy

AND

intestine flora OR gut microbiome OR gut microbiota OR intestinal microbiota OR gut microbial diversity OR gastrointestinal microbiome OR bacterial abundance OR bacterial richness OR bacterial diversity OR short chain fatty acid

AND

gut permeability OR gut integrity OR intestinal barrier OR tight junction OR digestive function OR gastrointestinal tract function OR digestive system disease OR constipation OR digestive system function disorder OR inflammatory bowel disease OR gut integrity OR bowel motion OR stool frequency OR abdominal distention OR gastrointestinal comfort OR gastrointestinal motility

**Supplementary Table 1.** Human population characteristics

| **Author** | **Year** | **Country** | **N** | **Sex (% female)** | **Age (years)** | **GI comp** | **GI inclusion criteria** |
| --- | --- | --- | --- | --- | --- | --- | --- |
| Ishikawa et al. (28) | 2003 | Japan | 21 | 47.6 | Range: 39-60 | IBD | UC |
| Beniwal et al. (29) | 2003 | USA | 202 | 56.9 | Range: 19-94 | FGID | AAD |
| Kato et al. (30) | 2004 | Japan | 20 | 50.0 | Mean: 32.0 | IBD | UC |
| Matsumoto et al. (31) | 2010 | Japan | 30 | 33.3 | Mean: 42.5 | FGID | FD |
| Søndergaard et al. (32) | 2011 | Denmark, Sweden | 52 | 75.0 | Range: 29-67 | IBS | IBS-SSI* |
| Marteau et al. (33) | 2013 | France | 530 | 100.0 | Range: 18-59 | FGID | Minor digestive symptoms |
| Tilley et al. (34) | 2014 | Belgium | 106 | NR | Range: 18-65 | FGID | Mild constipation |
| Veiga et al. (35) | 2014 | UK | 28 | 100.0 | Range: 20-69 | IBS-C | Rome III |
| Gomi et al.  (36) | 2015 | Japan | 27 | 13.3 | Range: 21-58 | FGID | Gastric symptoms |
| Liu et al.  (37) | 2015 | China | 118 | 50.8 | Range: 30-60 | FGID | FC (Rome III) |
| Thijssen et al. (38) | 2016 | NL | 80 | 33.9 | Range: 18-65 | IBS | Rome II |
| Le Nevé et al. (39) | 2019 | Sweden | 106 | 60.4 | Range: 18-65 | IBS | Rome III |
| Yilmaz et al.  (40) | 2019 | Turkey | 45 | 48.9 | Range: 19-68 | IBD | CD and UC |
| Li et al. (41) | 2020 | China | 20 | 85.0 | Mean: 47.0 | FGID | FC (Rome III) |
| Mokhtar et al. (42) | 2021 | Malaysia | 165 | 43.4 | Range: 20-50 | IBS | IBS-C (Rome III) |

N, sample size (completers); GI comp, gastrointestinal complications; GI, gastrointestinal, USA, United States of America; UK, United Kingdom; NL, The Netherlands; NR, data not reported; IBD, inflammatory bowel disease; FGID, functional gastrointestinal disorder; IBS, irritable bowel syndrome; IBS-C irritable bowel syndrome with constipation; UC, ulcerative colitis; AAD, antibiotic-associated diarrhoea; IBS-SSI, irritable bowel syndrome symptom severity scale; FC, functional constipation; CD, Crohn’s disease; FD, functional diarrhoea.

*IBS-SSI score >40cm along any scale.

**Supplementary Table 2.** Animal population characteristics

| **Author** | **Year** | **Country** | **N** | **Species** | **Sex** | **Age (wks)** | **GI comp** | **Comp induced** |
| --- | --- | --- | --- | --- | --- | --- | --- | --- |
| Uchida 2005 (43) | 2005 | Japan | NR | Sprague-Dawley rats | M | 1 | UC | TNBS |
| Veiga et al. (44) | 2010 | USA | 31 | TNFR1/p55  ^-/-^ mice | F | 8 | UC | TRUC |
| Sprong et al. (45) | 2010 | NL | 48 | Wistar rats | M | 8 | UC | DSS |
| Lee et al. (46) | 2015 | USA | 48 | BALB/c mice | F | 5 | UC | DSS |
| Liu et al. (47) | 2017 | China | 144 | Kunming mice | M | 8 | FGID (FC) | LP |
| Sevencan et al. (48) | 2019 | Turkey | 54 | Wistar rats | M | 18 | UC | TNBS |
| Rabah et al. (49) | 2020 | France | 90 | C57BL6 mice | F | 8 | UC | DSS |
| Yan et al. (50) | 2020 | China | 40 | C57BL/6J mice | M | 8 | UC | DSS |
| Zhang et al. (51) | 2020 | China | 100 | C67BL/6J mice | M | 8 | IBD | DSS |
| Feng et al. (52) | 2022 | China | 32 | Wistar rats | M | 7 | IBD | DSS |
| Yang et al. (53) | 2022 | China | 40 | Kunming mice | M | NR | FGID (AAD) | AB |

N, sample size; wks, weeks; GI comp, gastrointestinal complication; Comp, complication; NR, data not reported; M, male; F, female; USA, United States of America; NL, Netherlands; IBD, inflammatory bowel disease; UC, ulcerative colitis; TNBS, trinitrobenzene sulfonic acid; TNFR1/p55^-/-^, tumour necrosis factor receptor type 1 (p55); TRUC, T-bet^-/-^ RAG2^-/-^; DSS, dextran sodium sulphate; FGID, functional gastrointestinal disorder; LP, loperamide; BALB/c, AAD, antibiotic-associated diarrhoea; AB, antibiotic.

**Supplementary Table 3.** Test food nutritional information (human studies)

| **Author** | **Year** | **Test food** | **Intervention duration** | **Intervention**  **dose (daily)** | **Physical**  **structure** | **Fat**  **content (g/100g)** | **Protein content (g/100g)** | **CHO**  **content (g/100g)** | **Fermentation strain** |
| --- | --- | --- | --- | --- | --- | --- | --- | --- | --- |
| Matsumoto (31) | 2010 | Fermented milk | 4 weeks | 80mL | Liquid | 0.125 | 1.250 | 11.750 | *L.casei* strain Shirota |
| Tilley (34) | 2014 | Fermented milk | 8 weeks | 65mL | Liquid | 0.004 | 1.300 | 18.000 | L.casei strain Shirota |
| Liu (37) | 2015 | Yogurt | 7 weeks | 110mL | Gel/viscoelastic | 2.909 | 2.727 | 11.800 | *L. bulgaricus, S. thermophilus* |

CHO, carbohydrate; *L.casei* strain Shirota, *Lactobacillus casei* strain Shirota; *L.bulgaricus, Lactobacillus bulgaricus; S. thermophilus; Streptococcus thermophilus.*

**Supplementary Figure 1.** Risk of bias (human studies)
